# The Parkinsonian Gait Spatiotemporal Parameters Quantified by a Single Inertial Sensor before and after Automated Mechanical Peripheral Stimulation Treatment

**DOI:** 10.1155/2015/390512

**Published:** 2015-10-01

**Authors:** Ana Kleiner, Manuela Galli, Maria Gaglione, Daniela Hildebrand, Patrizio Sale, Giorgio Albertini, Fabrizio Stocchi, Maria Francesca De Pandis

**Affiliations:** ^1^Department of Electronics, Information and Bioengineering, Politecnico di Milano, 20133 Milano, Lombardia, Italy; ^2^Movement Analysis and Neuroscience-Neurological Rehabilitation Laboratories, University of Health Sciences of Porto Alegre, 90050-170 Porto Alegre, RS, Brazil; ^3^IRCCS San Raffaele Pisana Tosinvest Sanitá, 00163 Roma, Lazio, Italy; ^4^San Raffaele Cassino Hospital Tosinvest Sanitá, 03043 Roma, Lazio, Italy; ^5^UNIMED Hospital, 13500-391 Rio Claro, SP, Brazil

## Abstract

This study aims to evaluate the change in gait spatiotemporal parameters in subjects with Parkinson's disease (PD) before and after Automated Mechanical Peripheral Stimulation (AMPS) treatment. Thirty-five subjects with PD and 35 healthy age-matched subjects took part in this study. A dedicated medical device (Gondola) was used to administer the AMPS. All patients with PD were treated in off levodopa phase and their gait performances were evaluated by an inertial measurement system before and after the intervention. The one-way ANOVA for repeated measures was performed to assess the differences between pre- and post-AMPS and the one-way ANOVA to assess the differences between PD patients and the control group. Spearman's correlations assessed the associations between patients with PD clinical status (H&Y) and the percentage of improvement of the gait variables after AMPS (*α* < 0.05 for all tests). The PD group had an improvement of 14.85% in the stride length; 14.77% in the gait velocity; and 29.91% in the gait propulsion. The correlation results showed that the higher the H&Y classification, the higher the stride length percentage of improvement. The treatment based on AMPS intervention seems to induce a better performance in the gait pattern of PD patients, mainly in intermediate and advanced stages of the condition.

## 1. Introduction

The most typical gait pattern of Parkinson's disease (PD) is a short-stepped shuffling gait. It is characterized by reduced stride length and walking speed [1, 2]. These gait disorders worsen progressively, as the disease advances, and are related to the risk of falling among the Parkinsonians [3]. Therefore, it is not surprising that gait impairment in PD is the major contributor to decreased patients' quality of life [4].

The management of PD was traditionally centered on drug therapy, with levodopa being its “gold standard” treatment [5]. Several studies have demonstrated the ability of levodopa to decrease stride length and improve walk speed [6]. However, as the disease progresses, chronic levodopa treatment can be associated with response decrease and with development of motor complications, including wearing-off episodes and dyskinesia [5].

To reduce these motor fluctuations, new treatments based on peripheral stimulation of the sensory-motor system, called bottom-up stimulation, have been inspiring new rehabilitation approaches in PD [7, 8]. Recently, new approaches have been developed to recover the gait impairment such as the Automated Mechanical Peripheral Stimulation (AMPS) treatment [9, 10]. The AMPS is delivered by a dedicated device, known as Gondola (Gondola Medical Technologies SA, Switzerland), and consists in the application of a pressure via rounded stimulation tips in the four areas to be stimulated (two in each foot, which are the head of the big toe and the first metatarsal joint).

Stocchi et al. [9] evaluated the change in gait and the clinical status of 18 patients with PD after 6 sessions of a treatment based on AMPS. The study results indicate that the AMPS treatment has positive effect on bradykinesia and allows the improvement of walking velocity. Furthermore, AMPS has a positive effect on the step and stride length and on walking stability, measured as the increase in stride length and the reduction of double support time during walk. These results are consistent, and the results of improvement were measured via clinical scales.

Also recently, Galli et al. [10] evaluated a group of PD patients before and after AMPS evaluated with the Timed Up and Go (TUG) test, a widely used clinical performance-based measure of fall risk, measured with inertial sensors. The AMPS treatment improves the walking stability and seems to reduce the risk of falls in patients with PD. After the AMPS patients performed the TUG test faster and improved some kinematic parameters as the velocity to stand up from a chair and to sit down.

Based on these findings, the current study aims to evaluate the impact of the AMPS in functional abilities, measured with gait spatiotemporal parameters based on a single inertial wearable sensor. Recently, wireless inertial sensing devices are being developed also for the assessment of spatial-temporal parameters in unobstructed environment outdoors, thus overcoming the typical limitations of measurements in indoor laboratory settings. Several applications in the rehabilitation and recovery of patient mobility have been already reported by using these devices [11–14], more specific in patients with PD [5, 15–17].

The aim of this study was to assess and to quantify if the AMPS is capable of promoting changes on spatiotemporal parameters of PD gait. More specifically, this paper aims to assess the associations of the patients' clinical status with the percentage of improvement of the gait variables (stride length, velocity, cadence, and propulsion) after AMPS. The hypothesis of this study is that the AMPS stimulation improves the spatiotemporal gait of patients with PD, and the more compromised the patient is, the more benefits he/she will have after the bottom-up rehabilitation.

## 2. Methods

### 2.1. Participants

The Parkinson group (PD) consisted in 35 patients affected by Parkinson's disease. PD was diagnosed based on clinical criteria [18, 19], dopamine transporter (DaT) scans, and/or magnetic resonance imaging. All these patients are similar in terms of disease duration and are free of peripheral sensory neuropathy and other disorders based on their reported histories, symptoms, physical examinations, and clinical tests. Patients with liver, kidney, lung, or heart diseases, diabetes, or other causes of autonomic dysfunction were not included in the study.

The characteristics of the considered subjects are summarized in Table 1. The control group (CG) consisted in 35 healthy adults with the average characteristics in Table 1.

The study has been approved by the Ethics Research Committee of the IRCCS San Raffaele Institute. The trial was registered online at ClinicalTrials.gov (identifier number NCT01815281). All procedures were explained to the participants and were carried out with their adequate understanding, after receiving their written informed consent.

### 2.2. Experimental Procedures

During all intervention PD patients were in off phase, after an overnight withdrawal of all anti-Parkinsonian treatments.

#### 2.2.1. The Automated Mechanical Peripheral Stimulation (AMPS)

**Figure 1:**
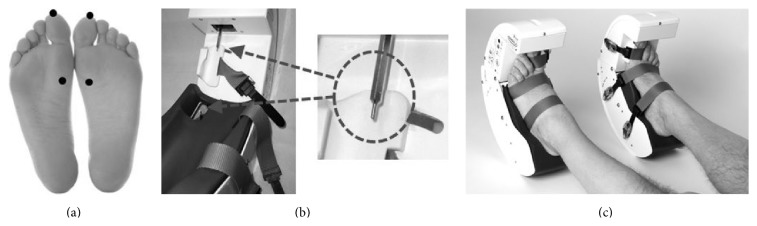
The device used for the AMPS treatment: (a) the specific points of feet stimulation; (b) the two moving steel bars; (c) patient positioning.

The treatment consists in the application of a pressure via rounded stimulation tips in four specific target areas in patient's feet (Figure 1(a)). To perform this mechanical stimulation, a dedicated medical device (Gondola, Gondola Medical Technologies, Lugano, Switzerland) was used to deliver the AMPS (Figure 1(b)). The system consists of feet supports (left and right) with electrical motors which activate two actuated steel bars with a 2 mm diameter; the motor-activated stimulators apply a mechanical pressure in two specific areas of each foot: on the head of the hallux, left and right, and on the 1st metatarsal joint, left and right.

Before treatment, the device needs to be adjusted to the patient's feet (Figure 1(c)): an inner sole of the correct size is inserted in each unit (left and right) to accommodate the feet; then the feet are inserted in the two units and tied up, using three straps per foot; after that, correct length steel bars are mounted on the axis of the electrical motors. The next step consists in positioning the motors that are mounted on adjustable platforms in order to make the steel bars interact with the areas to be stimulated (head of the hallux and first metatarsal joint of both feet). Once the device is adjusted, the excursion of the four motors (which work independently from each other) is programmed (using a remote control), aiming to apply the correct pressure stimulation on each area. The pressure of stimulation, always applied in a range of 0.3–0.9 N/mm^2^, is set for each subject upon appearance of the monosynaptic reflex in the Tibialis Anterior muscle by the detection of a liminaris contraction while applying pressure in the contact areas.

Once the pressure value has been set using this procedure, the value is recorded to administer the AMPS. This preparatory procedure requires approximately 10 minutes.

The treatment consists in 4 cycles; one cycle includes a stimulation of the 4 target areas requiring 24 seconds, whereas the overall 4-cycle treatment lasts for a total of 96 seconds. During the AMPS treatment, patients lay down (Figure 1(c)). At the end of the AMPS stimulation, both units of the device are removed from the feet of the patient; this final action is very easy and fast (less than 1 minute). This link shows images of a pre- and post-AMPS patient's gait (https://www.youtube.com/watch?v=deHFpt5gk3A&feature=youtu.be).

#### 2.2.2. The Inertial Sensor

The single inertial sensor is a wireless inertial sensing device (GSensor, BTS Bioengineering S.p.A., Italy) which provides acceleration along three orthogonal axes: anteroposterior, mediolateral, and superoinferior. Acceleration data were transmitted via Bluetooth to a PC and processed using dedicated software (BTS G-STUDIO, version: 2.6.12.0).

The portable GSensor consists in a wireless network of inertial sensors for human movement analysis. The sensors are controlled by a data logger unit (up to 16 elements), a ZigBee radio type communication. Each sensor is sized 62 mm × 36 mm × 16 mm, weighs 60 g, and is composed of a 3-axis accelerometer (max range ± 6 g), a 3-axis gyroscope (full scale ± 300°/s), and a 3-axis magnetometer (full scale ± 6 gauss). This sensing device is calibrated with the gravitational acceleration immediately after its manufacturing process. Only one sensor was used during this work. It was attached to the subjects' waists with a semielastic belt, covering the L4-L5 intervertebral space. The acceleration was analyzed about the three orthogonal anatomical axes: the anterior-posterior, mediolateral, and vertical axes.

The reference coordinate frame had the *z*-axis oriented to the front, *x*-axis oriented vertically upward, and *y*-axis orthogonal to the other two, towards the right. This motion analysis was performed with a sensitivity for the F4A accelerometer of 3G and a sampling frequency of 50 Hz. Acceleration data were transmitted via Bluetooth to a PC and processed with the use of dedicated software (BTS G-STUDIO, version: 2.6.12.0), which automatically provides the parameters described next.

All study participants were asked to walk at a self-selected speed along a pathway. Then, from the collected acceleration signals, the following typical spatial-temporal gait parameters were obtained:

- Stride length [m], the distance between two consecutive heel strikes of the same foot.
- Stride length/height [%], the stride length normalized by subject height.
- Speed [cm/s], the average instantaneous speed within the gait cycle as integration of acceleration.
- Cadence [strides/min], the number of strides in a minute.
- Propulsion [m/s²], the anterior-posterior acceleration peak during the lower limb swing phase.

### 2.3. Statistical Analysis

For statistical analysis, the data were first tested for normality with the Kolmogorov-Smirnov test. Because all the behavioral data exhibited normal distributions, parametric statistics were applied. The one-way ANOVAs (*α* < 0.05) were applied to compare the anthropometric data (i.e., age, body mass, and height) between the PD group and the CG. Furthermore, this test was applied to compare the differences between the right and the left lower limbs of the PD group and the CG. Once no significant differences were found between the right and left limbs, the left limb was selected to represent the CG and PD bodies for all gait variables comparisons.

Then, the described parameters were computed for each participant and for each trial, and significant values and standard deviations of all indexes were calculated for each group. After verifying that the parameters were normally distributed by means of Kolmogorov-Smirnov test, the one-way ANOVA for repeated measures (*α* < 0.05) was performed to assess the differences between pre- and post-AMPS; also, the one-way ANOVA for independent measures (*α* < 0.05) was performed to assess the differences between PD before and after AMPS and control group.

Next, Spearman's correlations (*α* < 0.05) were used to assess the associations between the Hoehn & Yahr (H&Y) [20] patient with PD clinical status and the percentage of improvement of the gait variables (stride length, velocity, cadence, and propulsion) after AMPS. The interpretation of the correlation degree is as follows: 0.9 to 1 indicated a very high correlation; 0.7 to 0.9 indicated a high correlation; 0.5 to 0.7 indicated a moderate correlation; 0.3 to 0.5 indicated a low correlation; and 0 to 0.3 indicated little to no correlation. All tests were two tailed. SPSS (version 19, IBM, Armonk, New York, United States) was used to perform all statistical analyses.

## 3. Results

**Figure 2:**
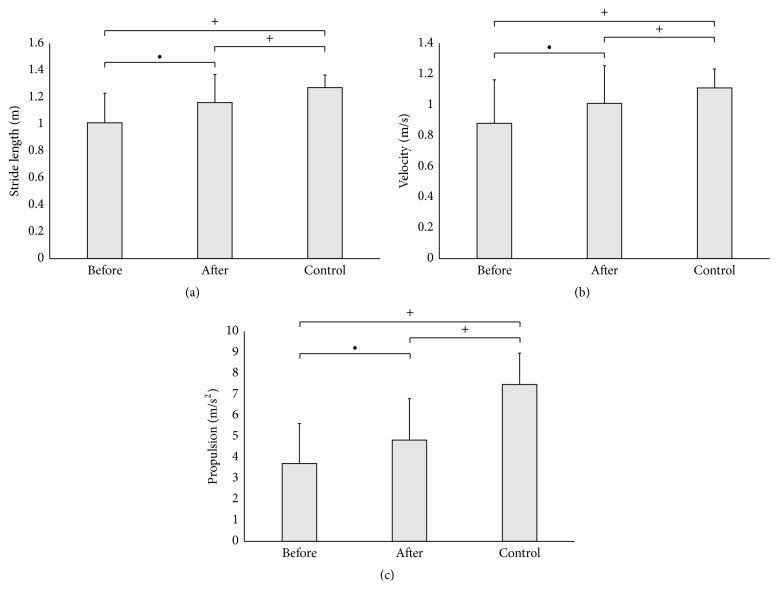
Significance and standard deviation of gait spatiotemporal parameters before and after AMPS: (a) stride length; (b) velocity; and (c) propulsion. *∙* = *P* < 0.05 between pre- and post-AMPS; + = *P* < 0.05 between PD and control group.

Figure 2 illustrates the spatiotemporal gait parameters results before and after AMPS. The patients with PD post-AMPS treatment presented longer stride length (Figure 2(a)); higher gait velocity (Figure 2(b)); and higher propulsion (Figure 2(c)).

**Figure 3:**
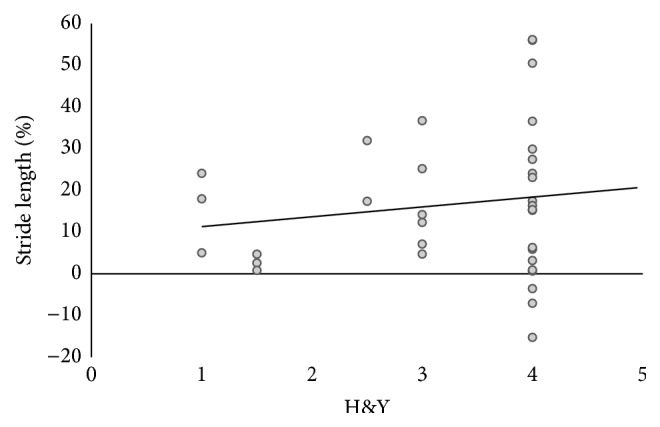
Correlation observed between the PD clinical status (H&Y) and the stride length percentage of improvement (stride length %) after AMPS.

For the 35 PD patients evaluated; 57.14% had H&Y stage 4; 20% had H&Y stage 3; 5.71% had H&Y Stages 2 and 5; 8.57% had H&Y Stages 1 and 5; and 8.57% had H&Y stage 1.  Figure 3 illustrates a significant and high positive correlation observed between the clinical status of the PD patients (H&Y) and the stride length percentage of improvement after AMPS (*ρ* = 0.733; *P* = 0.013). The more compromised the PD patient, the higher the percentage of the stride length improvement after AMPS intervention.

## 4. Discussion

The aim of this study was to evaluate the effect of AMPS treatment in PD subjects using a single inertial sensor to quantify the gait spatiotemporal parameters. Supporting our hypothesis, this study's results indicated that the AMPS stimulation improves the spatiotemporal gait parameters (stride length, walking velocity, and propulsion) of patients with PD and showed high correlation between the patient clinical status (H&Y) and the stride length percentage of improvement after AMPS.

Before AMPS, the spatiotemporal data acquired by a single inertial sensor were in accordance with previous studies [5], and the PD patients in the off stage of levodopa presented lower stride length and slower walking than the aged matched control groups.

Notwithstanding remaining in the “off medication” state, after one intervention with AMPS the PD group had an improvement of 14.85% in the stride length; 14.77% in the gait velocity; and 29.91% in the gait propulsion. The AMPS treatment seems to generate a more stable walking pattern in PD patients, reducing the well-known gait impairment that is typical of Parkinson's disease, mainly in off stages. Stocchi et al. [9] and Galli et al. [10] support these findings.

Moreover, the results of this study give a new insight of the AMPS as an effective therapy for the well-being of PD patients that helps improving their dynamic balance, especially in compromised clinical status patients. The correlation results show that the more severe the impairment of the PD patient, the higher the percentage of stride length improvement induced by the AMPS intervention.

The study has some limitations. The relatively small number of participants studied resulted in limited strength of the statistical findings. However, it documents the use of a new approach for the PD patient rehabilitation: the AMPS treatment applied via dedicated portable device.

## 5. Conclusion

The treatment based on AMPS induces a better performance in the gait pattern of PD patients. The obtained results showed that the AMPS treatment represents a promising rehabilitation. The results indicated that PD patients may be potential beneficiaries of the AMPS treatment once they face many neuromotor deficits, mainly in intermediate and advanced stages of the disease. These results are in agreement with our previous study done by a multifactorial quantitative laboratory. Moreover, the wearable devices are able to detect the typical motor fluctuations of PD patients after off levodopa and to document and quantify improvements following rehabilitation techniques such as the AMPS treatment.

## Figures and Tables

**Table 1:** Anthropometric characteristics.

Variables	Parkinson	Control group	*P*
Age (years)	68.15 ± 6.83	66.27 ± 6	0.419
Body mass (kg)	74.8 ± 12.54	73.22 ± 11.45	0.147
Height (cm)	162.73 ± 13.04	164.81 ± 10.10	0.315
H&Y	3.27 ± 1.09	—	—
UPDRS III	30.1 ± 10.4	—	—
Disease duration (years)	10.2 ± 6.3	—	—

## References

[1] Nemanich S. T., Duncan R. P., Dibble L. E. (2013). Predictors of gait speeds and the relationship of gait speeds to falls in men and women with parkinson disease. *Parkinson's Disease*.

[2] Morris M. E., Iansek R., McGinley J., Matyas T., Huxham F. (2005). Three-dimensional gait biomechanics in Parkinson's disease: evidence for a centrally mediated amplitude regulation disorder. *Movement Disorders*.

[3] Pickering R. M., Grimbergen Y. A. M., Rigney U. (2007). A meta-analysis of six prospective studies of falling in Parkinson's disease. *Movement Disorders*.

[4] Hobert M. A., Maetzler W., Aminian K., Chiari L. (2014). Technical and clinical view on ambulatory assessment in Parkinson's disease. *Acta Neurologica Scandinavica*.

[5] Curtze C., Nutt J. G., Carlson-Kuhta P., Mancini M., Horak F. B. (2015). Levodopa is a double-edged sword for balance and gait in people with parkinson's disease. *Movement Disorders*.

[6] Schaafsma J. D., Giladi N., Balash Y., Bartels A. L., Gurevich T., Hausdorff J. M. (2003). Gait dynamics in Parkinson's disease: relationship to Parkinsonian features, falls and response to levodopa. *Journal of the Neurological Sciences*.

[7] De Nunzio A. M., Grasso M., Nardone A., Godi M., Schieppati M. (2010). Alternate rhythmic vibratory stimulation of trunk muscles affects walking cadence and velocity in Parkinson's disease. *Clinical Neurophysiology*.

[8] Ebersbach G., Edler D., Kaufhold O., Wissel J. (2008). Whole body vibration versus conventional physiotherapy to improve balance and gait in Parkinson’s disease. *Archives of Physical Medicine and Rehabilitation*.

[9] Stocchi F., Sale P., Kleiner A. F. R. (2015). Long-term effects of automated mechanical peripheral stimulation on gait patterns of patients with Parkinson's disease. *International Journal of Rehabilitation Research*.

[10] Galli M., Kleiner A. F. R., Gaglione M. (2015). Timed up and go test and wearable inertial sensor: a new combining tool to assess change in subject with Parkinson's disease after automated mechanical peripheral stimulation treatment. *International Journal of Engineering and Innovative Technology*.

[11] Rueterbories J., Spaich E. G., Larsen B., Andersen O. K. (2010). Methods for gait event detection and analysis in ambulatory systems. *Medical Engineering & Physics*.

[12] Schwesig R., Leuchte S., Fischer D., Ullmann R., Kluttig A. (2011). Inertial sensor based reference gait data for healthy subjects. *Gait and Posture*.

[13] Godfrey A., Conway R., Meagher D., O’Laighin G. (2008). Direct measurement of human movement by accelerometry. *Medical Engineering & Physics*.

[14] Horak F., King L., Mancini M. (2015). Role of body-worn movement monitor technology for balance and gait rehabilitation. *Physical Therapy*.

[15] Del Din S., Godfrey A., Rochester L. (2015). Validation of an accelerometer to quantify a comprehensive battery of gait characteristics in healthy older adults and Parkinson's disease: toward clinical and at home use. *IEEE Journal of Biomedical and Health Informatics*.

[16] Esser P., Dawes H., Collett J., Feltham M. G., Howells K. (2012). Validity and inter-rater reliability of inertial gait measurements in Parkinson's disease: a pilot study. *Journal of Neuroscience Methods*.

[17] Salarian A., Russmann H., Vingerhoets F. J. G. (2004). Gait assessment in Parkinson's disease: toward an ambulatory system for long-term monitoring. *IEEE Transactions on Biomedical Engineering*.

[18] Gelb D. J., Oliver E., Gilman S. (1999). Diagnostic criteria for Parkinson disease. *Archives of Neurology*.

[19] Nutt J. G., Wooten G. F. (2005). Diagnosis and initial management of Parkinson's disease. *The New England Journal of Medicine*.

[20] Hoehn M. M., Yahr M. D. (1967). Parkinsonism: onset, progression and mortality. *Neurology*.

